# Effect of hormonal therapy on the otoconial changes caused by estrogen deficiency

**DOI:** 10.1038/s41598-022-27240-5

**Published:** 2022-12-30

**Authors:** Takahiro Nakata, Masahiro Okada, Eriko Nishihara, Aoi Ikedo, Sawa Asoh, Taro Takagi, Naohito Tokunaga, Naohito Hato, Yuuki Imai

**Affiliations:** 1 Department of Otolaryngology, Head and Neck Surgery, Ehime University Graduate School of Medicine, Shitsukawa, Toon, Ehime 791-0295 Japan; 2 Department of Otolaryngology, Ehime Prefectural Niihama Hospital, Niihama, Japan; 3 Division of Integrative Pathophysiology, Proteo-Science Center, Ehime University, Toon, Japan; 4 Division of Medical Research Support the Advanced Research Support Center, Ehime University, Toon, Japan; 5 Department of Pathophysiology, Ehime University Graduate School of Medicine, Toon, Japan; 6 Division of Laboratory Animal Research, Advanced Research Support Center, Ehime University, Toon, Japan

**Keywords:** Neuroscience, Neurology

## Abstract

Benign paroxysmal positional vertigo (BPPV) is associated with menopause and/or osteopenia. Morphological changes in the otoconial layer have been reported after ovariectomy (OVX). Moreover, hormone replacement therapy decreases BPPV risk. However, knowledge concerning the effect of hormonal therapy on the otoconial changes caused by estrogen deficiency is limited. We aimed to examine the effect of hormonal therapy on otoconial changes caused by estrogen deficiency. We hypothesized that hormonal therapy could reduce otoconial changes caused by OVX. Eight-week-old C57BL/6 mice were divided into four groups: sham operation with implantation of vehicle (sham + v), OVX with implantation of vehicle (OVX + v), OVX with implantation of estradiol (E2) (OVX + E2), and OVX with implantation of raloxifene (RAL) (OVX + RAL) groups. Otoconial layer volume was measured by micro-CT at 4 weeks after OVX or the sham operation. The otic bullae were removed; immunohistochemistry was performed for estrogen receptor alpha and 4-hydroxynonenal. Otoconial layer volume was significantly higher in the OVX + v than in the sham + v group. E2 and RAL significantly reduced these changes in the endometrial layer. The staining of estrogen receptor alpha and 4-hydroxynonenal were stronger in the OVX + v than in the sham + v group but equal in the sham + v, OVX + E2, and OVX + RAL groups. These results indicate that E2 and RAL are effective against morphological changes of the otoconial layer caused by estrogen deficiency via oxidative stress reduction.

## Introduction

Benign paroxysmal positional vertigo (BPPV) is a common vestibular disorder. A typical BPPV attack is triggered by horizontal or vertical head movement. Several patients claim that a severe vertigo attack occurs when lying down, turning over, or sitting up in bed. The cumulative incidence of BPPV during a lifetime is almost 10% at an age of 80 years^1^. The widely accepted etiology of BPPV is that the dislodged otoconial debris from the utricle floats into the endolymph and is trapped in the semicircular canal. The change in head position results in the movement of otoconial debris by gravity and follows an abnormal endolymph stream, leading to an inappropriate cupula signal, which finally gives rise to a vertigo attack. The natural course of BPPV without canal repositioning therapy results in spontaneous remission for several weeks^2^. The canalith repositioning procedure is often performed for patients with BPPV. The cure rate of the canalith repositioning procedure has been reported to be higher (94.2%) compared with that of no treatment (36.4%), and the relapse rate is 3.8% within 6 months^3^. Despite its short-term effect, it has been reported that 33.8–50% of patients with BPPV have a recurrence within several years^4–7^, and BPPV impairs the quality of life of patients^8^. However, there is no standard treatment for BPPV.

According to previous studies, BPPV is most common in middle-aged female individuals^9^, and hormone replacement therapy decreases its risk^10^. In addition, it has been reported that patients with BPPV tend to have low bone mineral density and low serum concentrations of vitamin D^6,11–16^, and those who have recurrence had a lower level of vitamin D than the non-recurrence group^6,12,15,17^. These studies suggest that BPPV is related to postmenopausal conditions or bone metabolism disturbance. In previous animal studies, bilateral ovariectomy (OVX), which induces estrogen deficiency and osteoporosis, caused morphological changes in otoconia^18–20^. It has also been reported that supplementation with phytoestrogen prevents otoconial changes induced by OVX^18^. Estrogen has been shown to have antioxidant properties^21^, while other studies have reported that BPPV is associated with oxidative stress^22–24^. However, no studies have investigated the effect of estrogen or osteoporosis drugs on changes in otoconia due to estrogen deficiency as well as the relationship between estrogen deficiency and oxidative stress in the utricle. Therefore, we aimed to examine this issue in this study.

Estrogen is a steroid hormone and acts as a transcription factor through estrogen receptor (ER), which is one of the nuclear receptors^25^. The administration of estrogen is applied to climacteric symptoms^26^. However, the administration of estrogen for the treatment of postmenopausal osteoporosis is controversial^27–29^. For such treatment, raloxifene (RAL) administration is widely provided. RAL is a selective ER modulator (SERM). Moreover, it is a non-steroidal agent that binds to ER^27^. Its action as an agonist or antagonist varies according to the organ tissue^30^. However, it remains unknown whether RAL acts as an agonist or antagonist in the inner ear. To examine this issue, in this study, we chose RAL for osteoporosis treatment. Our hypothesis was that estrogen or osteoporosis drugs could prevent the otoconial changes caused by estrogen deficiency via reducing oxidative stress. This study may provide useful information concerning the treatment options for preventing the occurrence of BPPV.

## Results

### Uterine weight

**Figure 1:**
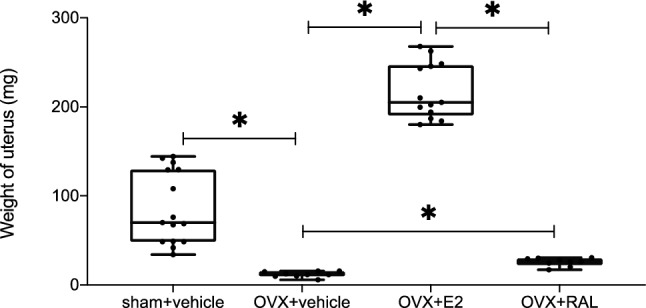
Weight of the uterus (each n = 9–15). The weight of the uterus decreased after ovariectomy (OVX). The administration of estradiol (E2) significantly increased the weight of the uterus relative to OVX with vehicle (*p* < 0.001). The uterus weight was significantly higher in the OVX + raloxifene (RAL) than in the OVX + v group (*p* = 0.002). (ANOVA: *p* < 0.001, *: *p* < 0.01).

Figure 1 shows the uterine weight measurements for each group at 4 weeks postoperatively. The uterine weights were 86.4 ± 39.4 mg, 12.0 ± 3.0 mg, 217.7 ± 30.1 mg, and 25.8 ± 4.4 mg, in the sham + vehicle (sham + v), OVX + vehicle (OVX + v), OVX + Estradiol (OVX + E2), and OVX + Raloxifene (OVX + RAL) groups, respectively. The uterine weight in the OVX + v group was significantly lower than that in the sham + v group (*p* < 0.001). The uterine weight in the OVX + E2 group was significantly higher than that in the OVX + v (*p* < 0.001) and OVX + RAL groups (*p* < 0.001). These data showed that OVX was performed correctly, the pellet of estradiol was absorbed by the mouse, and the pellet of raloxifene did not strongly affect the uterus.

### Bone mineral density

**Figure 2:**
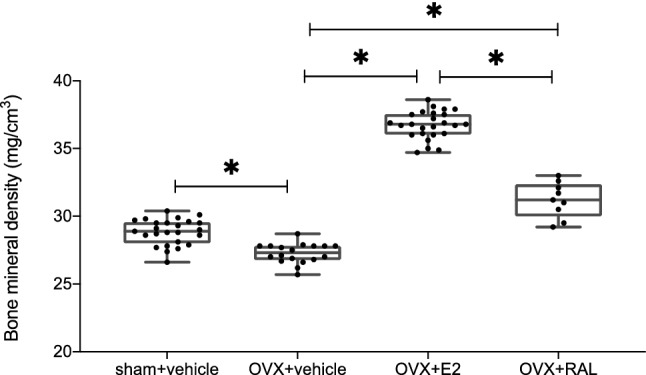
The bone mineral densities (BMDs) of the femurs measured by DEXA (each n = 9–25). The BMDs of the femurs were significantly lower in the OVX + v than in the sham + v group (*p* < 0.001). The administration of estradiol or raloxifene significantly increased the BMDs of the femurs relative to those in the OVX + v group (*p* < 0.001 and *p* < 0.001, respectively). (ANOVA: *p* < 0.001, *: *p* < 0.01).

Figure 2 shows the total bone mineral densities (BMDs) of the femurs in each group at 4 weeks postoperatively. The femoral BMDs were 28.8 ± 1.0 mg/cm^3^, 27.3 ± 0.7 mg/cm^3^, 36.7 ± 1.0 mg/cm^3^, and 31.2 ± 1.2 mg/cm^3^ for the sham + v, OVX + v, OVX + E2, and OVX + RAL groups, respectively. The BMD was significantly lower in the OVX + v than in the sham + v group (*p* < 0.001). The BMDs were significantly higher in the OVX + E2 and OVX + RAL groups than those in the OVX + v group (both *p* < 0.001). These data demonstrated that the administrations of estradiol and raloxifene were effective for bone metabolism.

### Volume of the otoconial layer

**Figure 3:**
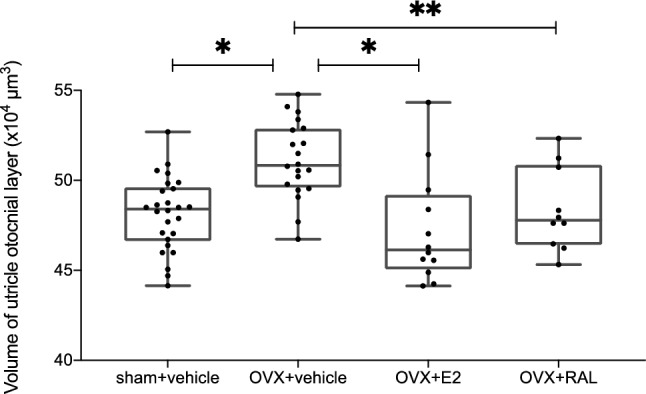
The volume of the utricle otoconial layer was measured using a micro-CT scan (each n = 10–26). The volume of the utricle otoconial layer in the OVX + v group was significantly higher than that in the sham + v group (*p* < 0.001). The volumes of the utricle otoconial layers in the OVX + E2 and OVX + RAL groups were significantly lower than those in the OVX + v group (*p* = 0.007 and *p* = 0.04, respectively) (ANOVA: *p* < 0.001, *: *p* < 0.01, **: *p* < 0.05).

Figure 3 shows the volume of the utricle otoconial layer obtained by micro-CT (µCT) for each group at 4 weeks postoperatively. The volumes of the utricle otoconial layer were 481,272.0 ± 19,998.2 µm^2^, 511,274.3 ± 20,755.1 µm^2^, 472,776.9 ± 29,765.6 µm^2^, and 483,702.3 ± 21,973.8 µm^2^ for the sham + v, OVX + v, OVX + E2, and OVX + RAL groups, respectively. The volume of the utricle otoconial layer was significantly higher in the OVX + v group than in the sham + v group (*p* < 0.001), whereas it was not significantly different in the sham + v, OVX + E2, and OVX + RAL groups.

### RNA-Seq analysis

**Figure 4:**
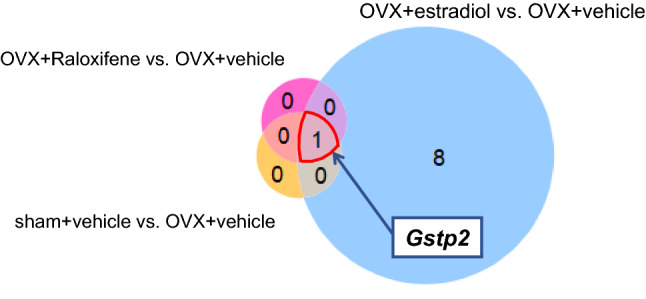
The Venn diagram for mRNA expression in the utricle of mice. The Venn diagram for mRNA expression difference in the utricle of the sham + vehicle, OVX + estradiol, and OVX + RAL groups compared to the OVX + vehicle group under a false discovery rate of 0.05 (each n = 5). Only the expression of *Gstp2* significantly decreased in the OVX + vehicle group compared with those of the other groups.

**Figure 5:**
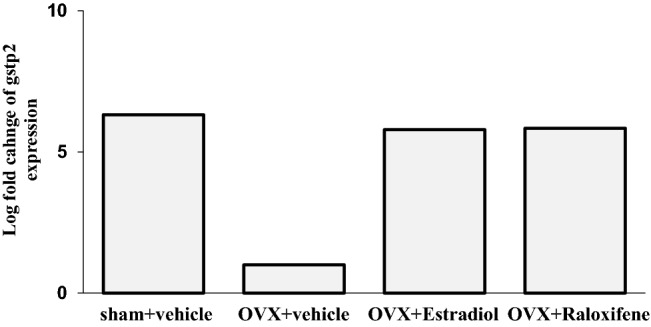
The expression of *Gstp2* in the utricle (each n = 5). The expression of *Gstp2* in the OVX + vehicle group was significantly lower than those in other groups (false discovery rate [FDR]-*p* < 0.001 vs. sham + vehicle group; FDR-*p* = 0.02 vs. OVX + estradiol group; FDR-*p* = 0.003 vs. OVX + raloxifene group).

Differential expression analysis of total mRNA in the utricle was performed. After obtaining the results of the volume of the otoconial layer, the data of the OVX + v group were compared with those of the sham + v, OVX + E2, OVX + RAL groups. In comparison between the OVX + E2 and OVX + v groups, the expression of *Gstp2* in the OVX + E2 group was significantly higher, and the expressions of *Orc8*, *Amd2*, *Mettl16*, *Slc35b1*, *Cog5*, *Wdr41*, *Sra1*, and *Stard8* in the OVX + E2 group were significantly lower. In comparison between the OVX + RAL and OVX + v groups and between the sham + v and OVX + v groups, the expression of *Gstp2* gene was found to be significantly higher in the OVX + RAL and sham + v groups. As aforementioned, the expression of *Gstp2* gene was significantly lower in the OVX + v group than in the other groups (Figs. 4 and 5). *Gstp2* codes glutathione S-transferase (GST) pi2. GST pi2 (GSTP) is a phase II detoxification enzyme that can detoxify reactive oxygen species or endogenous and exogenous toxic compounds^31^.

### Immunohistochemistry

**Figure 6:**
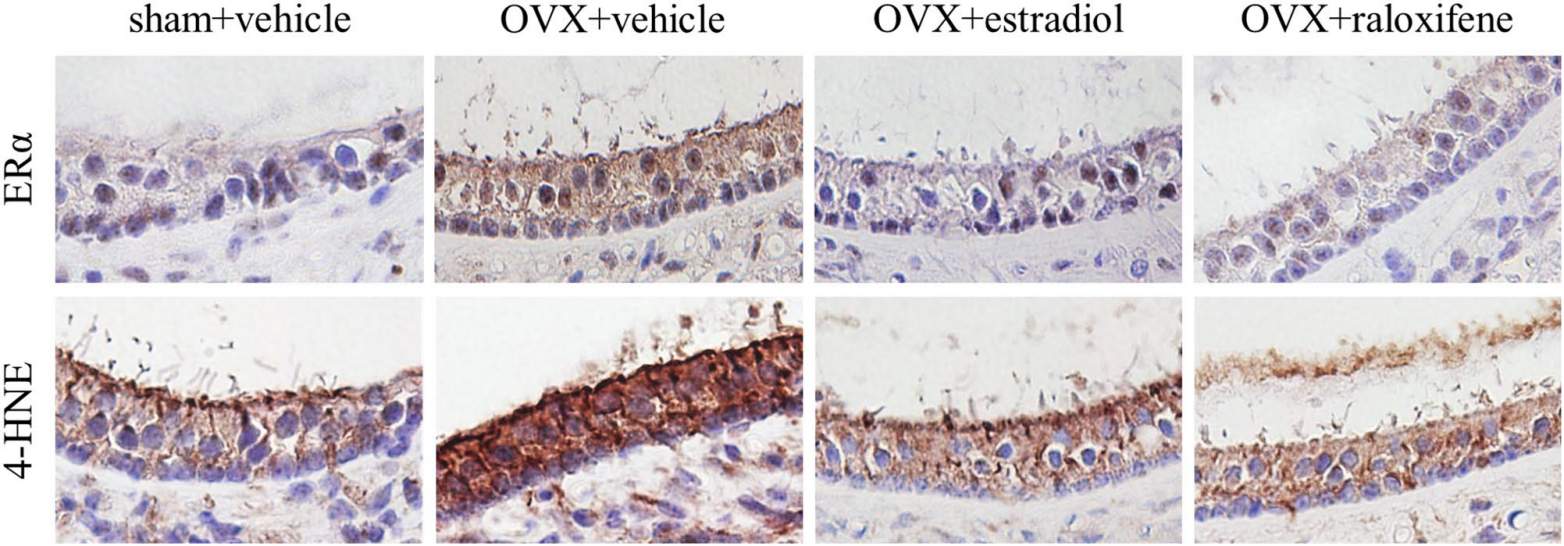
The immunohistochemistry of utricle. Staining of both estrogen receptor alpha (ERα) and 4-hydroxynonenal (4-HNE) were markedly stronger in the OVX + vehicle group than in the sham + vehicle group. Staining of ERα and 4-HNE in the OVX + estradiol and OVX + raloxifene groups were lower than those in the OVX + vehicle group and equal to those for the sham + vehicle group.

A higher intensity of the ER alpha (ERα) at the utricle was observed for the OVX + v group than for the sham + v group, and treatment with estradiol or raloxifene decreased it to the level observed for the sham + v group (Fig. 6). In addition, the intensity of 4-hydroxynonenal (4-HNE) was markedly higher in the OVX + V group than in the other groups. Especially, 4-HNE is a metabolite of lipid peroxidation and is known to be an oxidative stress marker^32^.

## Discussion

In this study, the volume of the utricle otoconial layer significantly increased by OVX, leading to estrogen deficiency. The administration of E2 or RAL prevented these morphological changes in the otoconial layer caused by OVX. RNA-seq analysis revealed that the expression of *Gstp2,* which encodes GSTP, significantly decreased in the OVX + v group. Immunohistochemical analysis showed that the expressions of ERα and 4-HNE were higher in the OVX + v group than in the other groups. These results suggest that the administration of E2 or RAL was effective in reducing the morphological changes in the utricle otoconial layer caused by estrogen deficiency by suppressing oxidative stress via ERs.

A previous work reported that OVX induced large and loose otoconia^19^ as well as higher volume of otoconial layer in the utricle^20^. Our findings of the OVX + v group support those of the aforementioned reports. It is notable that E2 and RAL suppress these changes of otoconial layer due to OVX. Although our data showed the change of ERα expression, we did not measure ERß since the previous study showed that estrogen regulated the expression of ERα while ERß was stable in the inner ear^33^. However, it remains unknown how ERα and ERß work to maintain the structure of utricle. Further studies are required to investigate the association of estrogen deficiency with ERα or ERβ.

The RNA-seq analysis revealed that the expression of *Gstp2* was significantly lower in the OVX + v group than in the other groups (Figs. 4 and 5). *Gstp2* GSTP, a member of the GST family. GSTs are phase II enzymes that detoxify endogenous or exogenous xenobiotics to protect cells^31^. GSTs catalyze the conjugation of electrophiles and cause cellular dysfunction with glutathione. The generated glutathione conjugates are eliminated extracellularly through a transporter, which is a multidrug resistance protein that protects cells from damage^34,35^. In addition to this detoxification, GSTP has recently been reported to regulate stress kinase signaling pathways^36–38^. Jun-terminal kinase (JNK) is a representative stress kinase that is triggered by stress, such as oxidative stress, head shock, and inflammatory cytokines^39–41^. GSTP forms a complex with JNK while preserving the basic JNK activity. Oxidative stress, cytokines, or drugs lead to the decomposition of the GSTP-JUN complex and activation of JNK signaling, resulting in apoptosis or proliferation^36,39,42^. Muthusami et al. reported that OVX induces impairment of the antioxidant system, including GST^43^. Our results suggest that estrogen deficiency decreases GSTP expression, leading to intracellular accumulation of oxidative components.

The staining intensity of 4-HNE and ERα in the utricle was stronger in the OVX + v than in the other groups (Fig. 5). The strong intensity of ERα in the OVX + v group may represent feedback of estrogen deficiency. Especially, 4-HNE is a metabolite of lipid peroxidation and a well-known oxidative stress marker^32^. Previous studies have demonstrated that menopause induces an increase in oxidative stress and decreases antioxidative activity^43–49^. In addition, hormone replacement therapy^44–47^ or supplementation with antioxidant agents^48^ have been reported to prevent the adverse effects of OVX. It has also been reported that estrogen has antioxidant properties independent of the ER^21,50,51^. These studies have revealed a strong relationship between estrogen and oxidative stress. Some recent clinical studies have also shown that BPPV is associated with oxidative stress^22–24^. Based on the aforementioned, it is suggested that the control of oxidative stress may be a novel approach to BPPV, particularly for perimenopausal patients.

In this study, E2 and RAL were effective for the suppression of morphological changes in the otoconial layer. RAL, which is one of the SERMs, prevents unnecessary effects on the ER in the mammary gland or uterus^30^. Therefore, RAL is frequently used to treat menopausal osteoporosis^27,52^. RAL acts on the ER in bones as an agonist and in the mammary glands or uterus as an antagonist^49^. This can suppress the risk of estrogen-induced tumors^30^. It has been reported that the mechanism of this unique action of SERM could be explained by the differential expression of ER, differential conformation of ER on ligand binding, and differential expression and binding of coregulator proteins in target cells^53^. ER has been shown to widely exist in the inner ear^54^. However, its action on the ER in the inner ear is unknown. In this study, similar to E2, RAL prevented the morphological changes in the otoconial layer caused by OVX. Furthermore, immunohistochemistry results showed that the intensity of OVX + E2 was almost the same as that of OVX + RAL in the utricle. However, it remains unknown whether RAL acts directly on the inner ear. There is a possibility that the improvement in bone metabolism by the administration of RAL may indirectly alleviate the changes in the otoconial layer caused by estrogen deficiency. It has been reported that bone metabolism is related to glucose metabolism, lymphopoiesis, and fat metabolism via osteocytes or osteoblasts^55,56^. Some factors secreted by osteocytes or osteoblasts may also regulate otolith metabolism. Further studies are needed to identify whether SERMs directly or indirectly affect the inner ear.

This study has several limitations. First, as mice are not genetically monoclonal like cultured cells, there may have been false negative genes during the RNA analysis. In addition, a real-time PCR assay to validate altered gene expression was not performed. Second, it remains unclear how oxidative stress morphologically changes otoconia. It has been shown that otoconia are mainly composed of constituent proteins, anchoring proteins, and regulatory proteins^57^. Oxidative stress may also affect the expression of these proteins. Further studies are required to identify the molecular mechanism underlying the effect of oxidative stress on the otoconial structure or vestibular environment. Third, we assessed the volume of the utricle otoconial layer using only µCT and did not perform other techniques, including histopathology or scanning electron microscopy (SEM). We previously reported that the volume of the otoconial layer measured using µCT was significantly associated with the histopathological study results^20^. Therefore, the µCT results were considered reliable. However, as the voxel size in the µCT was 6 µm, SEM was needed for detailed observations. Fourth, this study did not consider the estrous cycle. Mice have a 4–5-day estrous cycle^58^. However, it has not been reported whether the estrous cycle influences the vestibular system. Future studies are required to confirm whether the estrous cycle influences the otoconial structure. In addition, we did not measure serum E2 as the cost of E2 measurement is high. Instead, we ascertain E2 deficiency indirectly by measuring the uterus weight and BMD. As both the uterine weight and BMD significantly decreased by OVX in this study, the OVX mice were thought to be E2-deprived. However, further studies are needed to include E2 measurement. Finally, the dose of E2 and RAL administered by pellet was higher than the dose clinically used^59,60^. In this study, pellets were used to administer E2 or RAL to prevent animals’ stress. However, further studies are needed to investigate whether lower doses of these drugs are effective for preventing the morphological changes of otoconial layer due to OVX.

In conclusion, estrogen deficiency led to morphological changes in the utricle otoconial layer, and the administration of E2 or RAL prevented these changes in the otoconial layer. The mechanism underlying the effects of E2 or RAL remains unknown. However, the results of this study suggest that E2 and RAL reduce oxidative stress. Further works are needed to investigate whether E2 or RAL reduce oxidative stress via ER.

## Material and methods

The study protocol was approved by the Ethics Committee for Animal Experiments of Ehime University (No. 05HI77-1). All methods were performed in accordance with the relevant guidelines and regulations. This study was conducted in accordance with ARRIVE guidelines (https://arriveguidelines.org).

### Experimental animals and medications

Female C57BL/6 J mice were purchased from Japan SLC (Shizuoka, Japan) and randomly divided into the following four groups: (1) sham-operated with vehicle pellet (sham + v group), (2) OVX with vehicle pellet (OVX + v group), (3) OVX with pellet releasing 17beta-estradiol (OVX + E2 group), and (4) OVX with pellet-releasing raloxifene (RAL), which is a SERM (OVX + RAL group). The mice were either sham-operated or ovariectomized at 8 weeks of age under anesthesia using intraperitoneal injections of medetomidine (0.3 mg/kg), midazolam (4 mg/kg), and butorphanol (5 mg/kg). In the procedure of OVX, we made a skin incision at the lower back and identified the peritoneum. Then, we performed dissection of the peritoneum, removed the ovary, and finally sutured the incision. In sham-operated mice, the ovaries were identified and not removed, and vehicle pellets were implanted subcutaneously immediately after OVX. In the OVX mice, the following pellets were implanted subcutaneously: vehicle pellet, slowly releasing pellet of estradiol (50 µg/60 days), or slowly releasing pellet of raloxifene (2.4 mg/60 days) (Innovative Research of America, Sarasota, FL, USA). After OVX or sham operation, the mice were returned to their cages until further testing. All mice were kept in the same environment at a temperature of 21–23 °C and with a 12/12 h light/dark cycle (lights on 7:00 am to 7:00 pm), and they had free access to standard chow and water, ad libitum, at the animal facility at Ehime University.

The mice were sacrificed at 4 weeks after the OVX or sham operation, considering the results of a previous study, in which the volume of otoconial layer significantly increased at 4 weeks after OVX^20^. They were deeply anesthetized with ketamine (200 mg/kg) and xylazine (20 mg/kg) and euthanized. The otic bullae were removed and fixed with a 4% paraformaldehyde solution for 24 h, decalcified with 0.1 M EDTA for 4 days, and embedded in paraffin. The total BMDs of the femurs were measured using dual-energy X-ray absorptiometry with a bone mineral analyzer (DCS-600EX, Aloka, Tokyo, Japan). The uterine weight was measured to examine the effects of OVX and medication.

### Evaluation of the otoconial layer

**Figure 7:**
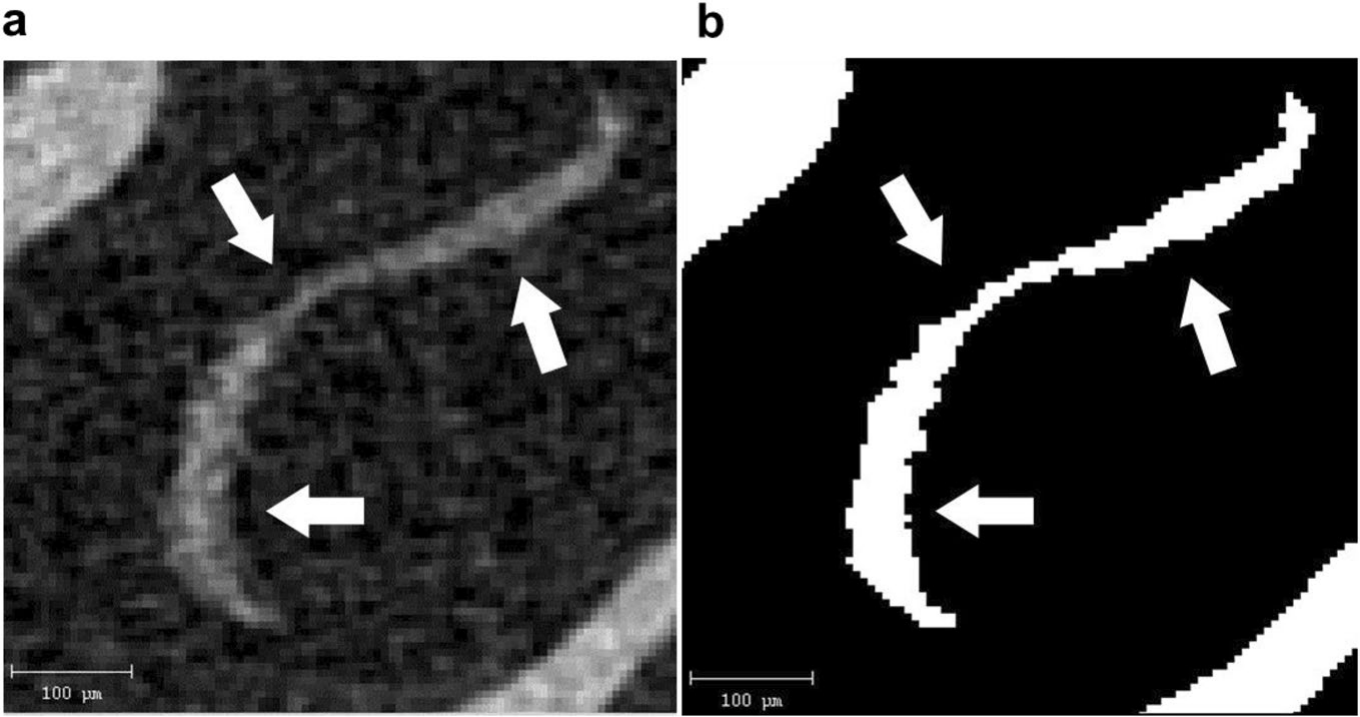
The micro-CT image of the utricle. (**a**) The original image of the utricle otoconial layer (white arrow) was obtained from a micro-CT scan. (**b**) The binarized image of the otoconial layer using Image J software. After binarization, the volume of the utricle otoconial layer was calculated using image J software.

The volume of the otoconial layer was assessed using µCT (Scanco Medical, Brüttisellen, Switzerland). We previously reported that the volume of the otoconial layer obtained from µCT scan images was correlated with that obtained in a histological study^20^. After sacrificing the mice under deep anesthesia, the otic bullae were removed and fixed with a 4% paraformaldehyde solution for 24 h. The otic bullae were post-fixed with 70% ethanol for 24 h, and µCT scanning using a Scanco Medical µCT35 System with an isotropic voxel size of 6 µm was performed. Scans were conducted with an X-ray tube potential of 70 kVp and an intensity of 114 µA, and the images of the utricle otoconial layer were analyzed using ImageJ software (US National Institute of Health, Bethesda, ML, USA) (Fig. 7). After binarization, the membranous structure of utricle otoconia was determined. The total areas of the utricle membranous structure from all slices for the four groups were compared.

### RNA-Seq analysis

After decapitation, the otic bullae were removed. The oval window was carefully fenestrated underwater, and the utricle was dissected. The utricle was rapidly stabilized using RNAprotect Tissue Reagent (#76,104, Qiagen, Hilden, Germany) and stored at 4 °C until RNA extraction. The total RNA was extracted from the utricle using the NucleoSpin RNA XS kit (#74,902.50, Macherey–Nagel, Waltham, MA, USA), according to the manufacturer’s instructions. RNA quality was checked using an Agilent 2100 Bioanalyzer (#G2939A, Agilent Technologies, Santa Clara, CA, USA), the RNA6000 pico kit (#5067–1513, Agilent Technologies), and the 2100 expert software (version B.02.07, Agilent Technologies). High-quality total RNA, which showed over 7 RNA integrity numbers (RIN), was used for further analysis, (average, 7.96; 7.30–8.24). The RNA-Seq library was constructed from 1 ng of total RNA using the QuantSeq 3’mRNA-Seq Library Prep Kit for Illumina (FWD) (#015.384, LEXOGEN, Vienna, Austria). The constructed library was sequenced using the NextSeq500 (Illumina) with NextSeq 5050/550 High Output Kit v2.5 (75 cycles) (#20,024,906, Illumina) at the Kazusa DNA Research Institute (Chiba, Japan). Sequence data were analyzed using the CLC Genomics Workbench (Qiagen). Briefly, the fastq was trimmed based on the guidelines of the library construction kit. Especially, the adaptor and polyA sequences and low-quality nucleotides at the 3’-end were removed. Trimming of the first 12 nucleotides at the 5’ end was performed, and short-length reads under 20 nucleotides were removed from the data set. The trimmed fastq was mapped to the Mus musculus (GRCm39.105) reference sequence using default settings. Differential expression analysis was performed by the CPM normalization method.

### Immunohistochemistry

After embedding in paraffin, sections of the otic bullae Sects. (4 µm) were prepared. They were subsequently deparaffinized and rehydrated. Antigen retrieval was performed in citraconic acid buffer (Immunosaver®, #097–06,192, Fujifilm Wako Pure Chemical Corporation) for 45 min in an autoclave at 95℃. Endogenous peroxidase activity was inhibited with 3% hydrogen peroxide for 15 min, and sections were blocked with an R.T.U. Animal Free Blocker and Diluent® (SP-5035, Vector Laboratories, Burlingame, CA, USA). After rinsing with phosphate buffer saline (PBS), the sections were incubated with rabbit polyclonal antibodies against ERα (1:200, #106,132-T08, Sino Biological) and 4-HNE (1:400, #bs-6313R, Bioss) overnight at 4℃. After subsequent rinsing with PBS once again, they were incubated with a secondary antibody (Histofine Simple Stain Mouse MAX-PO(R) #414,341, Nichirei Biosciences, Tokyo, Japan) for 1 h at room temperature and stained with the DAB chromogen for 10 min. After a final rinsing, counterstaining was performed using Mayer’s hematoxylin.

### Statistical analysis

The results are expressed as means ± standard deviations. The data of the groups were compared using ANOVA and the Steel–Dwass test with JMP for Macintosh (SAS Institute Inc., Cary, NC, USA). Statistical significance was set at *p* < 0.05. For the differential expression analysis of mRNA, the data of the OVX + v group were compared with those of the sham + v, OVX + E2, and OVX + RAL groups using t-test with CLC Genomics Workbench (Qiagen). FDP *p*-values < 0.05 were considered statistically significant.

## Data Availability

The datasets generated and/or analysed during the current study are available in the GEO repository, GSE216449 (https://www.ncbi.nlm.nih.gov/geo/query/acc.cgi?acc=GSE216449).
